# Genome-wide prediction using Bayesian additive regression trees

**DOI:** 10.1186/s12711-016-0219-8

**Published:** 2016-06-10

**Authors:** Patrik Waldmann

**Affiliations:** Department of Animal Breeding and Genetics, Swedish University of Agricultural Sciences (SLU), Box 7023, 750 07 Uppsala, Sweden

## Abstract

**Background:**

The goal of genome-wide prediction (GWP) is to predict phenotypes based on marker genotypes, often obtained through single nucleotide polymorphism (SNP) chips. The major problem with GWP is high-dimensional data from many thousands of SNPs scored on several thousands of individuals. A large number of methods have been developed for GWP, which are mostly parametric methods that assume statistical linearity and only additive genetic effects. The Bayesian additive regression trees (BART) method was recently proposed and is based on the sum of nonparametric regression trees with the priors being used to regularize the parameters. Each regression tree is based on a recursive binary partitioning of the predictor space that approximates an unknown function, which will automatically model nonlinearities within SNPs (dominance) and interactions between SNPs (epistasis). In this study, we introduced BART and compared its predictive performance with that of the LASSO, Bayesian LASSO (BLASSO), genomic best linear unbiased prediction (GBLUP), reproducing kernel Hilbert space (RKHS) regression and random forest (RF) methods.

**Results:**

Tests on the QTLMAS2010 simulated data, which are mainly based on additive genetic effects, show that cross-validated optimization of BART provides a smaller prediction error than the RF, BLASSO, GBLUP and RKHS methods, and is almost as accurate as the LASSO method. If dominance and epistasis effects are added to the QTLMAS2010 data, the accuracy of BART relative to the other methods was increased. We also showed that BART can produce importance measures on the SNPs through variable inclusion proportions. In evaluations using real data on pigs, the prediction error was smaller with BART than with the other methods.

**Conclusions:**

BART was shown to be an accurate method for GWP, in which the regression trees guarantee a very sparse representation of additive and complex non-additive genetic effects. Moreover, the Markov chain Monte Carlo algorithm with Bayesian back-fitting provides a computationally efficient procedure that is suitable for high-dimensional genomic data.

**Electronic supplementary material:**

The online version of this article (doi:10.1186/s12711-016-0219-8) contains supplementary material, which is available to authorized users.

## Background

The concept of genome-wide prediction (GWP) was introduced by Meuwissen et al. [1] and refers to the idea that regression coefficients of single nucleotide polymorphism (SNP) markers can be summed to provide overall breeding values that are used for selection purposes. In order to identify SNPs that affect the phenotype of interest, state of the art genome-wide marker data comprise several thousands, sometimes millions of SNPs. Since the number of individuals ($n$) is necessarily smaller than the number of SNPs ($p$), in the range of several hundreds to a few thousands, the consequence is a multivariate high-dimensional statistical issue that is often referred to as the $p$ ≫ $n$ problem [2]. Joint modeling of the effects of all SNPs through standard multiple regression is not feasible because of the $p$ ≫ $n$ problem. For example, when $p$ > $n$ the ordinary least squares estimator is not consistent and will considerably over-fit the data resulting in a low prediction accuracy [1]. Other problems with big genome-wide datasets include spurious random correlations, incidental endogeneity, noise accumulation, and measurement error [3]. Two popular statistical approaches to overcome some of these challenges are regularized regression and variable selection [4].

Several studies have evaluated the predictive abilities of different statistical and machine learning methods in genome-wide selection situations e.g. [5, 6], but relatively few studies have assessed both parametric and nonparametric methods under different genetic architectures. Howard et al. [7] assessed the performance of ten parametric and four nonparametric methods in terms of accuracy and mean squared error using simulated genetic architectures that consisted of purely additive or two-way epistatic interactions in an F_2_ population derived from crosses of inbred lines. They found that the parametric methods predicted phenotypic values less accurately when the underlying genetic architecture was entirely based on epistasis, whereas the parametric methods resulted in only slightly better predictions than nonparametric methods when the underlying genetic architecture was additive. However, they did not evaluate any regression tree method.

The classification and regression trees (CART) method was developed by Breiman et al. [8]. A regression tree consists of three components: a tree structure with internal nodes, decision rules and a set of terminal nodes (also denoted leaves). Each observation moves down a tree according to the binary decision rules contained at each internal node until it reaches a terminal node. The terminal nodes are parameterized such that each observation that is contained within a terminal node is assigned the same value. Tree size determines the complexity of the model and it needs to be tuned to reach the optimum size. Regression trees yield a flexible model that allows for nonlinearities and interaction effects in the unknown regression function, but single trees have problems with high variance, lack of smoothness and difficulty to capture additive structure [2].

The random forest (RF) method [9] is a collection of many trees, often hundreds to thousands, where the trees are constructed from nonparametric bootstrap samples of the original data. RF belongs to the category of randomized independent regression trees, where trees are grown independently and predictions are averaged to reduce variance. Instead of finding the best split rule at a tree node by using all the predictor variables, RF selects at each node of each tree a random subset of variables that are used as candidates to find the best split rule for the node. The idea behind this is to de-correlate trees so that the average over the forest ensemble will have a lower variance. Thus, for RF choices need to be made on the number of bootstrap samples and the number of sub-samples of predictors for the decision rules. RF can also select and rank variables through different variable importance measures, which make it an important tool for genomic data analysis and bioinformatics research [10, 11].

Chipman et al. [12] introduced a Bayesian version of CART (BCART), which samples trees from the posterior distribution of trees using Markov chain Monte Carlo (MCMC) by proposing a number of possible alterations to the current tree fit (e.g. growing or pruning a specific leaf node). MCMC tree sampling uses the same incremental moves that form the basis of CART. Unfortunately, this means that the chains tend to get stuck in locally-optimal regions of the tree-space. As an alternative, Chipman et al. [13] developed the Bayesian additive regression trees (BART) method, which replaces a single tree parameter target with the sum of many small trees. BART belongs to the family of approaches based on additive regression trees, where each consecutive tree fits the residuals that are not explained by the remaining trees. Hence, BART is a sum-of-trees method, but is conceptually different from the random sampling approach of RF. Over-fitting is controlled by three prior distributions that result in simpler tree structures and less extreme estimates at the leaves. Since BART mostly constructs very short trees, MCMC sampling is fast and mixes relatively well. Empirical studies have frequently shown that BART outperforms alternative prediction methods [14].

The purpose of this study was to review regression trees and RF, establish a connection between these methods and traditional genetics, introduce the BART methodology, and compare the prediction properties of BART with those of the LASSO, Bayesian LASSO (BLASSO), genomic BLUP (GBLUP), reproducing kernel Hilbert space (RKHS) and RF methods. We used simulated data as well as real pig data to compare methods.

## Methods

### Regression trees

Consider a response vector ${\mathbf{y}} = \left( {y_{1} , \ldots ,y_{n} } \right)^{T}$ of $n$ observations of a continuous trait and let the $p$ predictors of the SNP values $\left\{ {0,1,2} \right\}$ be collected in the $n \times p$ design matrix ${\mathbf{X}} = \left( {{\mathbf{x}}_{1} , \ldots ,{\mathbf{x}}_{p} } \right)$. A decision tree contains three parts, i.e. ${\mathbb{T}}$ = (⨅, *j*, *τ*). ⨅ is the structure of the tree with a finite collection of nodes where each node $\eta$ has one parent node (except for the root node which has no parents) and either zero or two children nodes. The nodes with zero children are denoted leaves or terminal nodes, and are located at the bottom of the tree. The nodes with children are called internal nodes and represent a binary split of the parent block, which is governed by a decision rule that is fully described by $j_{\eta }$, which denotes the splitting variable at node $\eta$, and $\tau_{\eta }$ which refers to the location of the split along variable $j_{\eta }$. When a decision tree is applied to a regression problem, it is usually referred to as a regression tree [17]. Each leaf node in a regression tree is associated with a real-valued parameter $\mu_{r}$ that is collected into a vector $\varvec{\mu}= \left\{ {\mu_{1} , \ldots ,\mu_{R} } \right\}$. Each data vector $\left\{ {y_{i} ,x_{i} } \right\}$, where $x_{i} = x_{i1} , \ldots ,x_{ip}$, is associated with a certain leaf node via the function through tree ${\mathbb{T}}$:

1 $$f\left( {y_{i} ,x_{i} ;{\mathbb{T}},\varvec{\mu}} \right) = \mu_{{{\text{leaf}}_{{\mathbb{T}}} \left( {x_{i} } \right)}} .$$

A regression tree for two SNPs and one phenotype, the genetic interpretation and response surface are described in Additional file 1.

### Random forests

Single regression trees are easy to construct and still relatively flexible, but there are some limitations. First, regression trees tend to have a high variance because of the binary splits and because the errors in the higher nodes are propagated downwards. Hence, a small change in the data may result in a very different tree structure, i.e. trees can be instable. Second, the terminal node surface is not smooth. This is a minor problem for SNP predictors that only have three possible values. However, it can be challenging in situations where other continuous predictors are included in the model. Third, the binary splits will favor a non-additive structure (see [2] for further details).

In order to address the problems described above, Breiman [9] proposed the random forests (RF) methodology. The main idea of RF is to fit regression trees to bootstrap samples of the original data, and then average the result. The trees are often grown until a minimum node size is reached and each tree is likely to have different split points and tree structures. For $b = 1, \ldots ,B$ bootstrap samples $\left\{ {{\mathbf{y}}_{\varvec{b}} ,{\mathbf{X}}_{\varvec{b}} } \right\}$, the *b*th regression tree function is trained as:

2 $$f_{b} \left( {\varvec{ } \cdot \varvec{ };{\mathbb{T}}_{b} ,\varvec{\mu}_{\varvec{b}} } \right),$$

and predictions for new test predictors ${\mathbf{X}}^{{*}}$ are performed:

3 $$\hat{y}^{{*}} = \frac{1}{B}\mathop \sum \limits_{b = 1}^{B} f_{b} \left( {{\mathbf{X}}^{{*}} ;{\mathbb{T}}_{b} ,\varvec{\mu}_{\varvec{b}} } \right) .$$

One of the key improvements in RF is the reduction in variance obtained by reducing the correlation between bootstrapped trees. This is achieved in the tree growing process by choosing a random set of variables *k* for each binary split that is smaller than the total number of variables, typically $k = \sqrt p , p/3$ or is inferred based on the minimum out-of-bag (OOB) error. OOB is the mean prediction error on each training sample $z_{i} = \left\{ {y_{i} ,x_{i} } \right\}$, using only the trees that do not have $z_{i} = \left\{ {y_{i} ,x_{i} } \right\}$ in their bootstrap sample [2].

### Bayesian additive regression trees

Chipman et al. [13] introduced the Bayesian additive regression tree (BART) method, which as RF is a sum-of-trees model, where each tree is constrained by three regularization Bayesian priors so that its size and effect are small. The BART model is defined as:

4 $${\mathbf{y}} = \mathop \sum \limits_{m = 1}^{M} f\left( {{\mathbf{X}};{\mathbb{T}}_{m} ,\varvec{\mu}_{m} } \right) + {\mathbf{e}},$$

where the residuals are normally distributed with an error variance, i.e. ${\mathbf{e}} \sim N\left( {0,\sigma_{e}^{2} } \right)$, and $M$ is the number of trees to be fitted. By assuming that the tree components $\left( {{\mathbb{T}}_{m} ,\varvec{\mu}_{m} } \right)$ are independent of each other and of $\sigma^{2}_{e}$, and that the terminal tree nodes of each tree are independent, it is sufficient to define three priors, $p\left( {{\mathbb{T}}_{m} } \right)$, $p\left( {\left. {\mu_{rm} } \right|{\mathbb{T}}_{m} } \right)$ and $p\left( {\sigma^{2}_{e} } \right)$.

$p\left( {{\mathbb{T}}_{m} } \right)$ consists of three parts, i.e. (1) the probability that a node at depth $d$ is non-terminal which is specified as $\alpha \left( {1 + d} \right)^{\beta }$; (2) a uniform prior over the variables that are assigned for the interior splitting nodes; and (3) a uniform distribution over the splitting rule assignment at each interior node conditional on the splitting variable. Chipman et al. [13] showed that good default choices are $\alpha = 0.95$ and $\beta = 2$.

The prior of the terminal node parameter conditional on the tree $p\left( {\left. {\mu_{rm} } \right|{\mathbb{T}}_{m} } \right)$ is the conjugate normal distribution $N\left( {\mu_{\mu } ,\sigma_{\mu }^{2} } \right)$. The hyperparameters $\mu_{\mu }$ and $\sigma_{\mu }^{2}$ of this distribution are chosen so that ${ \hbox{min} }( {\mathbf{y}} ) = M\mu_{\mu } - \kappa \sqrt M \sigma_{\mu }$ and ${ \hbox{max} }( {\mathbf{y}} ) = M\mu_{\mu } + \kappa \sqrt M \sigma_{\mu }$. Chipman et al. [13] suggested an approach where ${\mathbf{y}}$ is rescaled so that ${ \hbox{min} }( {\mathbf{y}} ) = - 0.5$ and ${ \hbox{max} }( {\mathbf{y}} ) = 0.5$, and set $\mu_{\mu } = 0$ and $\kappa \sqrt M \sigma_{\mu } = 0.5$. Moreover, $\kappa = 2$ seems to provide a good default choice that appropriately shrinks the terminal node parameters $\mu_{rm}$ towards zero. Larger values of $\kappa$ and $M$ result in more regularization of $\mu_{rm}$.

The prior of the residual variance $p\left( {\sigma^{2}_{e} } \right)$ is the conjugate inverse scaled Chi square distribution $\sigma^{2}_{e} \sim\nu \lambda_{\sigma } /\chi_{\nu }^{2}$. The hyper-parameters $\nu$ and $\lambda_{\sigma }$ are chosen based on an upper bound of the residual standard deviation $\hat{\sigma }_{e}$. Chipman et al. [13] proposed two ways of estimating $\hat{\sigma }_{e}$, but only the approach that uses the sample standard deviation of ${\mathbf{y}}$ is possible in the $p$ ≫ $n$ setting. The value of $\nu$ should be between 3 and 10, and the value of $\lambda_{\sigma }$ to locate the *q*th quantile of the prior should be set at $\hat{\sigma }_{e}$. The default values are $\nu = 3$ and $q = 0.9$. The number of trees $M$ also needs to be set. Although it would be possible to estimate the optimal number of trees by assigning a hyper-prior to this number, Chipman et al. [13] recommended not doing this because it increases the computational load considerably. Based on simulated examples, they show that $M = 200$ provides very good prediction performance. An alternative is to choose the hyperparameters based on cross-validation.

### BART MCMC algorithm and posterior prediction

An MCMC algorithm for BART can be constructed based on Gibbs sampling with some Metropolis–Hastings steps [13]. Start by defining ${\mathbb{T}}_{ - m}$ as the set of all trees except tree ${\mathbb{T}}_{m}$, and $\varvec{\mu}_{ - m}$ as the set of all terminal node parameters except $\varvec{\mu}_{m}$. For each MCMC iteration, the Gibbs sampler draws successively from the following conditional distributions:

5 $$p\left( {\left. {{\mathbb{T}}_{m} ,\varvec{\mu}_{m} } \right|{\mathbb{T}}_{ - m} ,\varvec{\mu}_{ - m} ,\sigma_{e} ,{\mathbf{y}}} \right) \quad {\text{for}}\quad m = 1, \ldots ,M,$$

and

6 $$p\left( {\left. {\sigma_{e} } \right|{\mathbb{T}}_{1} , \ldots ,{\mathbb{T}}_{M} ,\varvec{\mu}_{1} , \ldots ,\varvec{\mu}_{M} ,{\mathbf{y}}} \right).$$

The $M$ draws from (5) rely on the calculation of the partial residuals based on the fit that excludes the *m*th tree $r_{m} = {\mathbf{y}} - \mathop \sum \nolimits_{{l \ne \varvec{m}}} f\left( {{\mathbf{X}};{\mathbb{T}}_{l} ,\varvec{\mu}_{l} } \right)$ and then, in turn, samples from:

7 $$p\left( {{\mathbb{T}}_{m} \left| {r_{m} ,} \right.\sigma_{e} } \right),$$

and

8 $$p\left( {\varvec{\mu}_{m} \left| {{\mathbb{T}}_{m} ,r_{m} ,} \right.\sigma_{e} } \right).$$

This algorithm is known as Bayesian backfitting [18]. In order to draw the trees in (7), a Metropolis–Hastings step is needed. The algorithm proposes new trees based on four possible changes of the current tree. The first move consists in growing a terminal node with probability $p = 0.25$, the second move in pruning a pair of terminal nodes with $p = 0.25$, the third move in changing an internal node with $p = 0.4$, and finally the fourth move in swapping a rule between parent and child with $p = 0.1$.

In (8), each of the entries in $\varvec{\mu}_{m}$ is sampled from a normal distribution, i.e. $\mu_{rm} \sim N\left( {0,0.5/\left( {\kappa \sqrt M } \right)} \right)$ where the default value of $\kappa$ is as mentioned above. Finally, the residual variance is drawn from a scaled inverse Chi square distribution, $\sigma^{2}_{e} \sim {\text{Scale}} - {\text{inv}} - \chi_{\nu }^{2} \left( {\nu ,\lambda_{\sigma } } \right),$ where the default value of $\nu$ is equal to 3 and $\lambda_{\sigma }$ is chosen to locate the 0.9 quantile of the prior at $\hat{\sigma }_{e}$.

The MCMC algorithm induces a sequence of $t = 1, \ldots ,T$ posterior draws:

9 $$f^{t} ( \cdot ) = \mathop \sum \limits_{m = 1}^{M} f\left( { \cdot \,;{\mathbb{T}}_{m}^{t} ,\varvec{\mu}_{m}^{t} } \right),$$

which can be used to perform mean predictions of new test data ${\mathbf{X}}^{{*}}$:

10 $$\hat{y}^{{*}} = \frac{1}{T}\mathop \sum \limits_{t = 1}^{T} f^{t} \left( {{\mathbf{X}}^{{*}} ; \cdot } \right).$$

### Evaluation of predictions

Since the main goal of genomic prediction is to predict the future phenotypes based on available genotype and phenotype data, the full dataset was divided into training and test datasets. The training dataset was used to learn the model parameters, which thereafter predict the phenotypes of the test dataset ($\hat{y}^{{*}}$). To find the best model, the mean squared prediction error (${\text{MSPE}}$) was then calculated as ${\text{MSPE}} = \frac{1}{ntest}\sum\nolimits_{1}^{ntest} {\left( {\hat{y}^{{*}} - {\mathbf{y}}_{{\varvec{test}}} } \right)^{2} }$. For the simulated QTLMAS2010 dataset, the 2326 individuals of generations 1–4 were used as training data and the 900 individuals of generation 5 were used as test data. This strategy corresponds to the two-generation cross-validation approach [19]. The real dataset of Cleveland et al. [16] was randomly divided into five different cross-validation sets that each comprised 70 % of training data and 30 % of test data, and the ${\text{MSPE}}$ was averaged over these cross-validation sets. This approach is an example of repeated random sub-sampling validation [19]. Predictions were obtained for the LASSO (using the glmnet package; [20]), Bayesian LASSO (BLASSO), genomic BLUP (GBLUP), Gaussian process with radial basis function kernel (GPRBF) as an example of a reproducing kernel Hilbert space (RKHS) method (all three using the BGLR package; [21]), RF (using the randomForest package; [9]) and BART (using the BayesTree package; [13]) methods.

LASSO and RF analyses were run with the default settings of the glmnet and randomForest packages. The minimum mean squared error (minMSE) and minMSE + 1 standard error of minMSE backwards along the regularization parameter *λ* path (minMSE + 1SE), i.e. the largest λ-value such that the error is within one SE of the minimum, were used as stopping criteria for LASSO [20, 40]. The MCMC of the BART analysis was run for 75,000 iterations for all datasets. Visual inspection of the $\sigma_{e}^{2}$ parameter showed that convergence was usually reached after a few thousand iterations. Hence, the first 25,000 iterations were excluded as burn-in, and the remaining iterations were thinned to a final sample of 5000. The MCMC of the BLASSO, GBLUP and RKHS analyses were run for 60,000 iterations, with a burn-in of 10,000 and thinning of 10. The regression coefficients were obtained for the GBLUP and RKHS methods using $\hat{\beta } = {\mathbf{X}}^{T} {\varvec{\Sigma}}^{ - 1} \varvec{u}/p$, where ${\varvec{\Sigma}}$ is the genomic relationship matrix for GBLUP and the genomic kernel matrix for RKHS. $\varvec{u}$ is the vector with predicted genetic values [19].

The bandwidth parameter of the radial basis function (RBF) of the RKHS method was optimized by evaluating $h = \left\{ {0.05,0.1,0.25} \right\}$ for the QTLMAS2010 data and $h = \left\{ {0.1,0.5,1} \right\}$ for the Cleveland data. BART were optimized by evaluating the ${\text{MSPE}}$ over different combinations of hyperparameters. Since BART is computationally demanding, the choice of the values was restricted to $M = \left\{ {10,25,50,100,200,400,600} \right\}$, $\kappa = \left\{ {2,3,4,5} \right\}$ and $q = \left\{ {0.9,0.95} \right\}$ for the QTLMAS2010 datasets, and $M = \left\{ {100,200,300} \right\}$, $\kappa = \left\{ {3,4,5,6} \right\}$ and $q = \left\{ {0.9,0.95} \right\}$ for the Cleveland dataset. The number of trees for RF was optimized by evaluating $M = \left\{ {10,25,50,100,200,400,600} \right\}$ in the QTLMAS2010 datasets and $M = \left\{ {100,200,300,400,600,800} \right\}$ in the Cleveland dataset.

### Variable importance measures and inclusion proportion

It is possible to obtain different variable importance measures (VIMP). In the RF approach, there are several measures of variable importance. One common approach for regression trees is to calculate the decrease in prediction accuracy from the OOB data. For each tree, the OOB portion of the data is passed through the tree and the prediction error (MSPE_OOB_) is recorded. Each predictor variable is then randomly permuted and *j* new MSPE_OOB_ are calculated. The difference between the two are then averaged over all trees, and normalized by the standard deviation of the differences [2]. The variable showing the largest decrease in prediction accuracy is the most important variable. The result is often displayed in a variable importance plot of the top ranked variables, or in Manhattan type scatter plots of all variables.

BART uses a different approach where the selected variables are those that appear most often in the fitted sum-of-trees models of the MCMC chains. For each posterior draw, the function $f^{t} ( \cdot )$ calculates the variable inclusion proportion (VIP) of all splitting rules that use variable *j* as $\pi_{j}^{t}$ and then the average as $\nu_{j} = \frac{1}{T}\sum\nolimits_{t = 1}^{T} {\pi_{j}^{t} }$. It should be noted that this approach depends on *M* and irrelevant predictors can get mixed with relevant predictors when *M* is very large [13].

### QTLMAS2010 simulated data

This data was initially created for the QTLMAS2010 workshop [15]. The simulated pedigree was founded by 20 individuals i.e. five males and 15 females and includes 3226 individuals across five generations. The pedigree structure was created by assuming that each female mates with only one male (mostly with males from their own generation) and gives birth to approximately 30 progeny. Five autosomal chromosomes were simulated, each about 100 Mb long. The biallelic SNP data was simulated using a neutral coalescent model. The algorithm produced 10,031 SNPs, including 263 monomorphic and 9768 biallelic SNPs. Mean LD ($r^{2}$ calculated from unphased genotypes) between adjacent SNPs with a minor allele frequency (MAF) higher than 0.05 was equal to 0.100 (SD = 0.152).

The continuous quantitative trait used in this study was determined by 37 quantitative trait loci (QTL), including nine known genes and 28 random genes. All QTL were modelled as additive effects, except for two pairs of epistatic QTL and three paternal imprinting QTL. The known genes were selected based on their high level of polymorphism and high linkage disequilibrium (LD) with SNPs. All known QTL had an additive effect of +3 (i.e. half the difference between the mean effects of homozygotes). The random genes were drawn from the simulated SNPs (excluding those on chromosome 5) and their effects were sampled from a truncated normal distribution, $N\left( {0,10} \right)$. They were selected if the absolute value of their additive effect was less than 2, i.e. the additive effects of the random genes ranged from −1.98 to 1.93. The two epistatic pairs of QTL were located on chromosomes 1 and 2, respectively, and determined by four controlled additive QTL with an additional epistatic effect of 4 for the lowest homozygous pairs. The imprinting effect was equal to 3. Each simulated QTL was surrounded by 19 to 47 polymorphic SNPs (MAF > 0.05) that were located within a distance of 1 Mb from the QTL. Of these SNPs, 364 were in moderate to high LD with the QTL ($r^{2} > 0.1$). The narrow-sense heritability ($h^{2}$) was equal to 0.52 for males and 0.39 for females. SNPs with a MAF lower than 0.01 were discarded, but SNPs that deviated from Hardy–Weinberg equilibrium (HWE) were not removed because regression trees can handle non-linear relations. A final set of 9723 SNPs was available.

In order to also evaluate if BART can detect various forms of dominance and epistasis, a second simulated dataset was created based on the QTLMAS2010 data by adding effects at different loci on chromosome 5: (1) SNP 9212 was a dominant locus by setting a value of 5 and 5.01 for the effect of the heterozygous (Aa) and homozygous states (AA) (for numerical reasons), respectively; (2) SNP 9404 was an over-dominant locus by assigning values of 5, −0.01 and 0.01 to the heterozygous (Aa) and homozygous (aa) and (AA) states, respectively; (3) SNP 9602 was an under-dominant locus by assigning values of −5, −0.01 and 0.01 to the heterozygous (Aa) and homozygous (aa) and (AA) states, respectively; and (4) two SNPs 9694 and 9695 that had no additive effects were chosen to create an epistatic effect by assigning values of −0.01 and 0.01 to the homozygous aa and AA states, and a value of 5 to both AA homozygous states. Finally, the values of these new SNPs were summed to the original **y**-values.

### Real data

Cleveland et al. [16] published a pig dataset that comprised 3534 individuals with high-density genotypes and phenotype records, and estimated breeding values for five traits. Genotypes were obtained with the PorcineSNP60 chip, which after quality control yielded 52,842 SNPs. Missing genotypes were imputed using a probability score which results in non-integer values. SNPs with both known and unknown positions were included and imputed, but the map order was randomized and SNP identity was recoded. The number of SNPs was further reduced by applying a more stringent MAF (<0.01), which resulted in a final number of 50,276 SNPs.

Genotyped animals had phenotypes for five purebred traits (phenotypes from a single nucleus line), with heritabilities ranging from 0.07 to 0.62. For this study, we chose the trait that had a heritability of 0.38. This phenotype was corrected for environmental factors and rescaled by correcting for the overall mean. Individuals with missing phenotype data were removed and a final number of 3141 individuals was used.

## Results

### QTLMAS2010 data

For the original QTLMAS2010 dataset, the LASSO with the minMSE option was found to produce a ${\text{MSPE}}$ of 62.020, which was the lowest value of all six methods (Table 1). The second best ${\text{MSPE}}$ (62.595) was obtained with BART for the hyperparameters $M = 200$, $\kappa = 4$ and $q = 0.9$. The BLASSO, GBLUP and RKHS methods performed more or less equally with ${\text{MSPE}}$ of 66.209, 66.949 and 66.821, respectively. The lowest ${\text{MSPE}}$ (76.141) for the RF method was found for 400 trees. Hence, RF can be considered to perform considerably worse than all other methods in terms of prediction error when the majority of the genetic effects are additive.

**Table 1:** Mean squared prediction error (MSPE) for the LASSO, Bayesian LASSO (BLASSO), genomic BLUP (GBLUP), reproducing kernel Hilbert space (RKHS) regression, random forests (RF) and Bayesian additive regression trees (BART) methods evaluated on the simulated original QTLMAS2010 data

Method	Mean squared prediction error (MSPE)						
LASSO							
*minMSE*	*62.020*						
*minMSE* + *1SE*	63.404						
BLASSO	*66.209*						
GBLUP	*66.949*						
RKHS							
$h = 0.05$	66.910						
$h = 0.1$	*66.821*						
$h = 0.25$	67.200						
RF	*M* = 10	*M* = 25	*M* = 50	*M* = 100	*M* = 200	*M* = 400	*M* = 600
	82.108	79.772	77.794	77.274	77.149	*76.141*	76.419
BART	*M* = 10	*M* = 25	*M* = 50	*M* = 100	*M* = 200	*M* = 400	*M* = 600
$$q = 0.9$$							
$\kappa$ = 2	76.231	69.974	65.703	64.967	64.324	64.213	64.574
$\kappa$ = 3	71.325	68.537	66.755	63.772	62.782	62.919	63.476
$\kappa$ = 4	79.264	66.554	66.376	63.596	*62.595*	63.119	63.790
$\kappa$ = 5	72.344	70.608	65.467	62.705	62.715	63.997	64.982
$$q = 0.95$$							
$\kappa$ = 2	78.656	76.734	68.282	64.126	64.218	63.697	64.566
$\kappa$ = 3	74.893	68.379	64.858	63.762	62.884	63.108	63.402
$\kappa$ = 4	74.128	66.817	64.788	63.836	62.596	63.175	63.807
$\kappa$ = 5	76.757	66.284	64.512	62.648	62.823	63.912	64.976

The lowest MSPE obtained with each method is highlighted in italics. *M* is the number of trees for RF and BART, and $q$ and $\kappa$ are hyperparameters of the BART priors. The stopping criteria for the regularization coefficient *λ* in LASSO were obtained based on tenfold cross-validation both at minimum MSE and minimum MSE plus 1 standard error [42]

The analysis of the QTLMAS2010 dataset when dominance and epistatic effects are added resulted in an ${\text{MSPE}}$ of 64.353 for BART with hyperparameters $M = 100$, $\kappa = 4$ and $q = 0.9$ (Table 2). This is considerably better than the results with BLASSO (${\text{MSPE}}$ of 71.857), LASSO (minMSE option) (${\text{MSPE}}$ = 83.377), RKHS (MSPE = 91.852), GBLUP (${\text{MSPE}}$ = 92.296) and RF ($M = 600$) (${\text{MSPE}}$ = 99.836). These results show that BART can detect complicated non-additive genetic effects and accommodate these in the predictions of phenotypes.

**Table 2:** Mean squared prediction error (MSPE) for the LASSO, Bayesian LASSO (BLASSO), genomic BLUP (GBLUP), reproducing kernel Hilbert space (RKHS) regression, random forests (RF) and Bayesian additive regression trees (BART) methods evaluated on the simulated QTLMAS2010 data when dominance and epistatic effects were added

Method	Mean squared prediction error (MSPE)						
LASSO							
*minMSE*	*83.377*						
*minMSE* + *1SE*	84.832						
BLASSO	*71.857*						
GBLUP	*92.296*						
RKHS							
$h = 0.05$	92.361						
$h = 0.1$	*91.852*						
$h = 0.25$	91.906						
RF	*M* = 10	*M* = 25	*M* = 50	*M* = 100	*M* = 200	*M* = 400	*M* = 600
	107.908	105.123	100.784	101.992	100.327	100.900	*99.836*
BART	*M* = 10	*M* = 25	*M* = 50	*M* = 100	*M* = 200	*M* = 400	*M* = 600
$$q = 0.9$$							
$\kappa$ = 2	80.717	76.892	70.845	65.294	65.196	66.283	66.906
$\kappa$ = 3	79.277	72.720	67.061	65.120	64.943	65.542	66.593
$\kappa$ = 4	87.030	71.401	65.635	*64.353*	65.149	66.483	68.050
$\kappa$ = 5	79.249	71.243	67.748	64.741	65.611	68.290	70.510
$$q = 0.95$$							
$\kappa$ = 2	86.328	70.452	67.744	65.465	65.308	65.801	66.998
$\kappa$ = 3	76.438	69.833	67.123	65.522	65.045	65.513	66.601
$\kappa$ = 4	86.653	74.651	67.164	67.220	65.074	66.544	68.163
$\kappa$ = 5	90.456	69.571	65.085	66.086	65.790	68.298	70.566

The lowest MSPE obtained with each method is highlighted in italics. *h* is the bandwidth of the radial basis function kernel. *M* is the number of trees for RF and BART, and $q$ and $\kappa$ are hyperparameters of the BART priors. The stopping criteria for the regularization coefficient λ in LASSO were obtained based on tenfold cross-validation both at minimum MSE and minimum MSE plus 1 standard error [42]

**Fig. 1:**
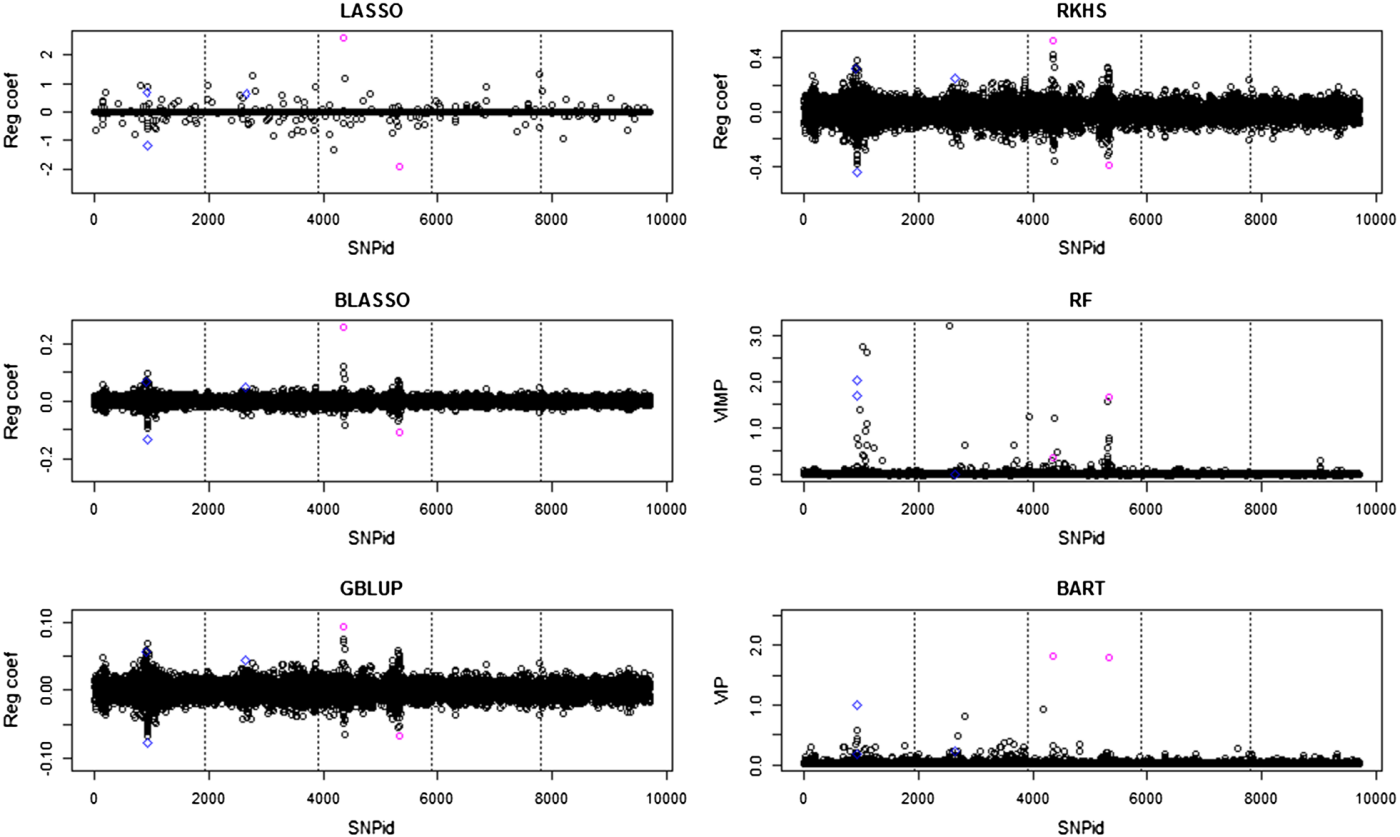
Manhattan plots of the penalized regression coefficients from the LASSO, BLASSO, GBLUP and RKHS methods, VIMP (percent decrease in MSE) for RF, VIP (average number of node splits per iteration) for BART from the analyses of the original QTLMAS2010 dataset. *Dotted lines* delimit chromosomes; the major additive genetic effects on chromosome 3 are indicated by *magenta circles* and the epistatic loci on chromosome 1 by *blue diamonds*

The regression coefficient and variable importance plots in Fig. 1 show that all methods detect the two major additive loci on chromosome 3 in the original QTLMAS2010 dataset. However, LASSO, BLASSO, GBLUP and RKHS assign a negative effect to the second additive locus, and RF has difficulties in detecting the first additive locus at the right position. The epistatic locus on chromosome 1 was also detected by all methods, but not the epistatic locus on chromosome 2. Neither of the imprinting effects were detected. Moreover, it is worth noting that BART seems to regularize very well, especially for loci on chromosome 5 that should have no genetic effects.

**Fig. 2:**
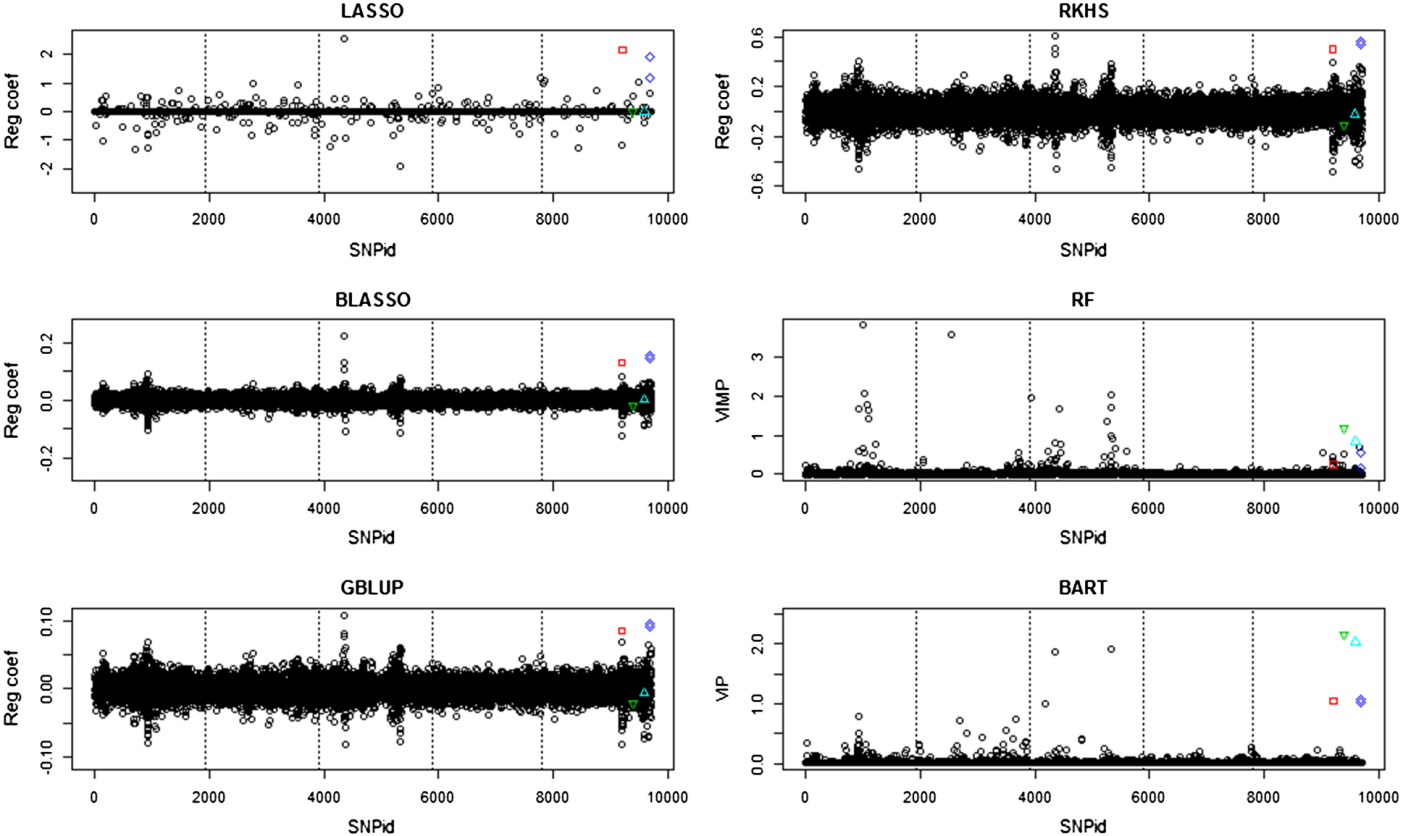
Manhattan plots of the penalized regression coefficients from the LASSO, BLASSO, GBLUP and RKHS methods, VIMP (percent decrease in MSE) for RF, VIP (average number of node splits per iteration) for BART from the analyses of the QTLMAS2010 dataset with non-additive genetic effects added on chromosome 5. *Dotted lines* delimit chromosomes. The dominant locus is indicated by a *red square*, the over-dominant locus by a *green upper triangle*, the under-dominant locus by a *cyan lower triangle*, and the epistatic loci by *blue diamonds*

Analyses of the QTLMAS2010 dataset for which the phenotype was constructed with additional dominance and epistatic loci are in Fig. 2 and show that LASSO, BLASSO, GBLUP and RKHS detect the first dominant locus and the two SNPs that form the epistatic locus, but neither the over- nor the under-dominant loci. Hence, these methods cannot handle dominance properly without the addition of matrices with a specified gene action. RF detected all non-additive effects, but they were not well-separated from adjacent noise. BART found all effects, with the over- and under-dominant loci having variable importance measures that are twice as high as the weakly dominant and epistatic loci. This corresponds very well with the fact that BART should split the over- and under-dominant loci into two nodes.

### Cleveland dataset

Analysis of the five random cross-validation partitions of the real pig dataset of Cleveland et al. [16] also showed that BART performed best with an average minimum ${\text{MSPE}}$ of 0.811 at hyperparameter values $M = 200$, $\kappa = 5$ and $q = 0.9$ (Table 3). The second best performance was obtained with the RF method with $M = 600$ yielding a ${\text{MSPE}}$ of 0.813, which is close to that obtained with BART. LASSO, BLASSO, GBLUP and RKHS performed worse with a ${\text{MSPE}}$ of 0.829, 0.821, 0.822 and 0.819, respectively. Hence, the ranking in terms of ${\text{MSPE}}$ of the six methods differs from the ranking based on the QTLMAS data.

**Table 3:** Mean squared prediction error (MSPE) for the LASSO, Bayesian LASSO (BLASSO), genomic BLUP (GBLUP), reproducing kernel Hilbert space (RKHS) regression, random forests (RF) and Bayesian additive regression trees (BART) methods evaluated on the pig PorcineSNP60 chip genotype data with one phenotype

Method	Mean squared prediction error (MSPE)					
LASSO						
*minMSE*	*0.829*					
*minMSE* + *1SE*	0.861					
BLASSO	*0.821*					
GBLUP	*0.822*					
RKHS						
$h = 0.1$	0.821					
$h = 0.5$	*0.819*					
$h = 1$	0.820					
RF	*M* = 100	*M* = 200	*M* = 300	*M* = 400	*M* = 600	*M* = 800
	0.819	0.820	0.815	0.817	*0.813*	0.813
BART	*M* = 100	*M* = 200	*M* = 300	*M* = 400	*M* = 600	*M* = 800
$$q = 0.9$$						
$\kappa$ = 3	0.822	0.820	0.821	–	–	–
$\kappa$ = 4	0.819	0.814	0.815	–	–	–
$\kappa$ = 5	0.814	*0.811*	0.812	–	–	–
$\kappa$ = 6	0.815	0.813	0.814	–	–	–
$$q = 0.95$$						
$\kappa$ = 3	0.826	0.820	0.821	–	–	–
$\kappa$ = 4	0.823	0.814	0.814	–	–	–
$\kappa$ = 5	0.815	0.812	0.812	–	–	–
$\kappa$ = 6	0.814	0.814	0.814	–	–	–

The estimates are the mean over five random cross-validation-folds with 70 % training and 30 % test partitions. The lowest MSPE obtained with each method is highlighted in italics. *h* is the bandwidth of the radial basis function kernel. *M* is the number of trees for RF and BART, and $q$ and $\kappa$ are hyperparameters of the BART priors. The stopping criteria for the regularization coefficient λ in LASSO were obtained based on tenfold cross-validation both at minimum MSE and minimum MSE plus 1 standard error [42]

**Fig. 3:**
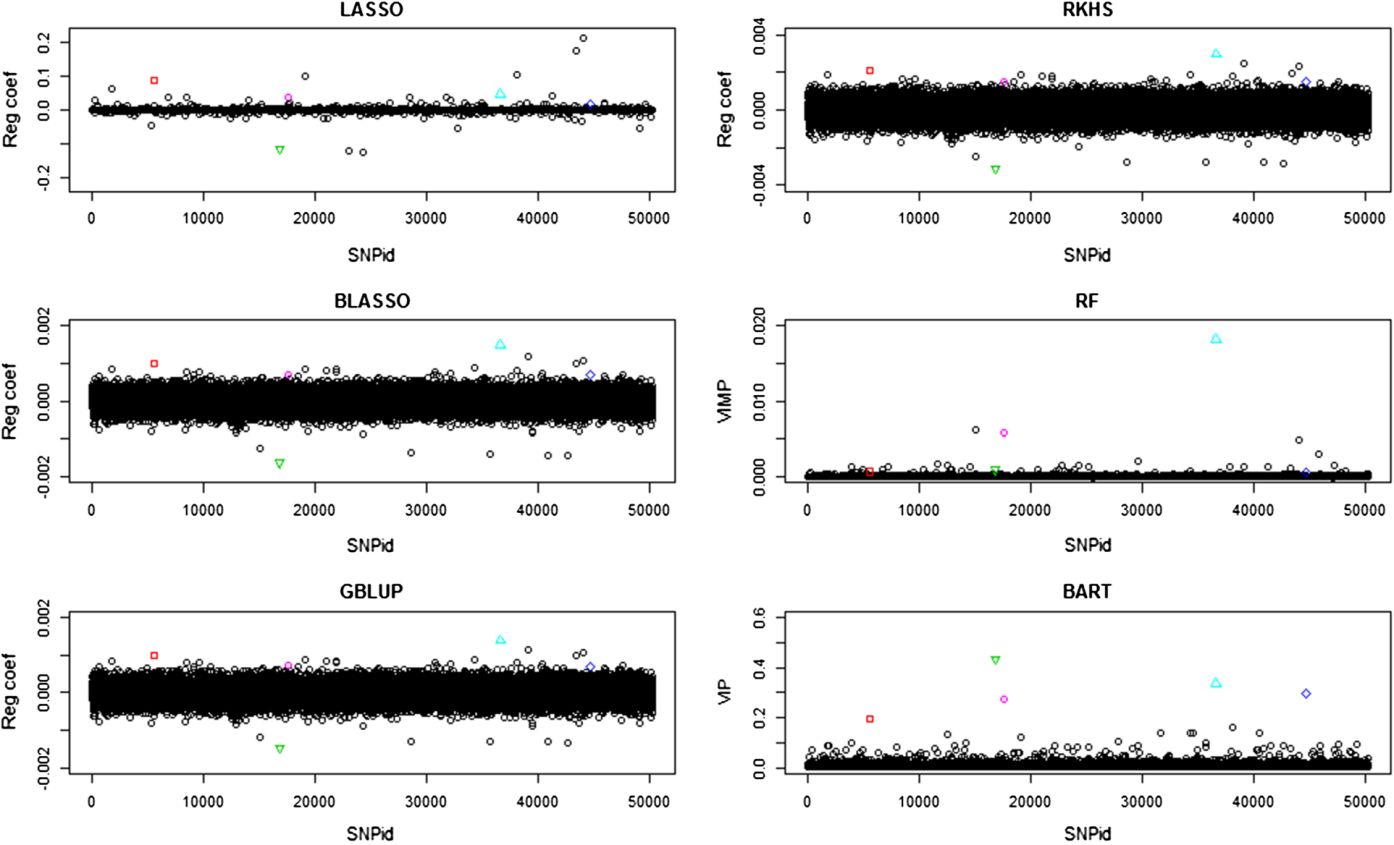
Manhattan plots of the penalized regression coefficients from the LASSO, BLASSO, GBLUP and RKHS methods, VIMP (percent decrease in MSE) for RF, VIP (average number of node splits per iteration) for BART from the analyses of the Cleveland pig dataset. The five most important SNPs in the BART analysis are highlighted in *different colors* for comparison with the other plots

Penalized regression coefficients of the LASSO and BLASSO methods, back-calculated regression coefficients of the GBLUP and RKHS methods, and variable importance measures of the RF and BART methods averaged over the five cross-validation partitions are in Fig. 3. The five highest ranked variables from the BART analysis have ${\text{SNPid}} = \left\{ {5583,16800,17552,36623,44686} \right\}$ and are marked in different colors. SNP 36623 was clearly detected by all methods. SNPs 5583 and 16800 were clearly separated in the LASSO, BLASSO, GBLUP, RKHS and BART analyses, but not so well in the RF analysis. SNP 15552 was more clearly separated in the RF and BART analyses than in the other analyses. SNP 44686 has a distinct effect only in the BART analysis.

## Discussion

The partitioning of genetic effects into additive and non-additive (dominance and epistasis) contributions has a long history in quantitative genetics [22]. The additive genetic effect is essentially the slope of a linear regression, whereas dominance refers to the deviation of the heterozygote from the linear additive genetic effect and epistasis to the interaction effects between different SNPs. Most GWP studies use statistical methods that aim at inferring linear additive genetic SNP effects [23]. This study is based on the prediction of phenotypes, not on the prediction of estimated breeding values (EBV). The reason for this choice is that most statistical methods used for the prediction of EBV enforce a linear additive genetic structure (e.g. BLUP) and therefore non-additive genetic GWP methods will be automatically disadvantaged when the statistical performance is evaluated using EBV, for example in the calculation of correlations between EBV and genomic EBV. Hence, there is an urgent need to switch focus from the restricted linearity assumptions in genome-wide studies to more realistic non-linear effects both within and between SNPs [19].

The RF methodology has been used in several genome-wide association studies (GWAS) and GWP studies. Cabras et al. [24] showed how RF can be successfully applied to discrete data on disease phenotypes for large-scale GWAS. González-Recio and Forni [25] evaluated the GWP properties of four methods using simulated discrete data and disease resistance data in pigs. They found that RF produced most consistent results with a very good predictive ability and outperformed other methods in terms of correct classification. Heslot et al. [26] used eight real datasets on plant breeding records to evaluate genomic selection properties of several statistical methods and found that RF together with the Bayesian LASSO and Bayesian variable selection methods performed best in terms of accuracy. Hence, it is somewhat surprising that RF performs worse than LASSO, BLASSO and GBLUP on the QTLMAS2010 data, but similar results have been obtained on large datasets in other studies [27]. The reason for this behavior is unclear, but we noted that RF did not detect the first additive locus on chromosome 3 properly. RF has been reported to be sensitive to highly correlated predictors [28]. One possible explanation is that highly correlated unimportant variables influence the building of trees and variable importance measures negatively.

The LASSO method sets unimportant variables to exactly zero and therefore provides an automatic variable selection procedure [2]. Bayesian LASSO can be implemented in different ways. The original version of Park and Casella [29] produces credible intervals that can be used for variable selection. Hans [30] developed a Gibbs sampling approach that is similar to the stochastic search variable selection method and can be used on relatively large scale $p$ ≫ $n$ data. Credible intervals can be calculated for SNP effects in the GBLUP and RKHS methods, but it is computationally demanding due to the need to perform back-calculations for each MCMC iteration. Variable selection in tree ensemble methods is more difficult because of their non-parametric nature and lack of formally defined test statistics. Regarding the RF method, Diaz-Uriarte and Alvares [31] proposed an iterative backward elimination procedure for selecting genes from microarray data. Genuer et al. [32] suggested a related heuristic rank-based method and Ishwaran et al. [33] described an approach for forest variable selection based on minimal depth, which is a measure of the distance of a variable relative to the root of the tree. Unfortunately, all these RF VIMP selection techniques have certain drawbacks when applied to large-scale data [10]. Recently, Bleich et al. [34] suggested three permutation-based procedures for variable selection in BART. The methods are based on permuting the response, fitting a BART to each permutation and calculating the three different test statistics of the VIMP. However, to obtain a reasonable amount of permutations, these procedures become computationally very demanding on large datasets and cannot be performed without applying parallelization [35].

It should also be noted that since ensemble regression tree methods are black-box approaches, it is rather difficult to evaluate the genetic effect of a given SNP. However, some tools are available to investigate how SNPs influence the prediction. Partial dependence plots provide a useful approximation to visualize non-linearity within and interaction between important variables [2]. The idea is to partition the predictors into a smaller subset ${\mathbf{X}}_{S}$ and its complement ${\mathbf{X}}_{C}$, where $S = \left\{ 0 \right\}$, $C = \left\{ {1,2} \right\}$ and $S \cup C$. Then, the partial dependence functions are estimated by $f_{S} \left( {{\mathbf{X}}_{S} } \right) = \frac{1}{n}\sum\nolimits_{i = 1}^{n} {f\left( {{\mathbf{X}}_{S} ,x_{iC} } \right)}$, where $x_{iC}$ are the values of individual $i$ in the complementary genotypes. The partial dependence functions represent the effect of ${\mathbf{X}}_{S}$ on $f\left( {\mathbf{X}} \right)$ after accounting for the average effect of ${\mathbf{X}}_{C}$ on $f\left( {\mathbf{X}} \right)$. The partial dependence functions can be evaluated for pairs of variables and thereby investigate epistatic effects. Unfortunately, similar computational difficulties apply to partial dependence plots as to variable selection, but it is likely that these problems will be solved in the near future.

The number of statistical machine learning methods has increased dramatically over the recent years [36, 37] and it is not possible to evaluate the prediction performance of all proposed methods. In this study, LASSO and its Bayesian variant were used as references with well-documented good prediction properties under linearity assumptions [38], GBLUP and RKHS methods were chosen based on their popularity in the GWP area [19], and the RF method was used as a well-performing frequentist reference for ensemble regression tree prediction [10, 11]. A natural extension would be to compare BART with other machine learning methods such as Bayesian stochastic processes [39], deep learning [40] and reinforcement learning [41].

## Conclusions

This study shows how the Bayesian additive regression tree method (BART) can be applied to large-scale genome-wide SNP data for the prediction of unknown phenotypes and detection of the SNPs that contribute information for the prediction. Since BART is based on an ensemble of regression trees, it is a non-parametric and non-linear method that has the important feature of being able to handle all types of genetic effects of SNPs in a very sparse way. Comparison of BART with the LASSO, BLASSO, GBLUP and RKHS methods using simulated data showed that the prediction error of BART under additive gene action was equally good or lower, and considerably better in the presence of dominance and epistasis. BART outperforms RF under all settings. Moreover, BART has the lowest prediction error of all methods for the analysis of real pig data, which indicates that non-additive gene action contributes to the analyzed phenotype. To date, no GWP applications have used BART. Hence, there is a need for further applications and evaluations of BART using data from different species.

## Additional file

10.1186/s12711-016-0219-8 Construction of regression trees from SNP data. This file describes how to build a regression tree for fictive data of two SNPs and one phenotype, and how to make the genetic interpretation of the resulting response surface.
