# Supplementary material for: Genome-wide prediction using Bayesian additive regression trees
Source: Genet Sel Evol. 2016 Jun 10;48:42. doi: 10.1186/s12711-016-0219-8 (PMC4901500; doi:10.1186/s12711-016-0219-8)
Supplement: Supplementary file 1 — 10.1186/s12711-016-0219-8 Construction of regression trees from SNP data. This file describes how to build a regression tree for fictive data of two SNPs and one phenotype, and how to make the genetic interpretation of the resulting response surface. [file 12711_2016_219_MOESM1_ESM.docx]

**Additional file 1**

The construction of a regression tree is best illustrated by considering a normal response $\mathbf{y}$ and two predictor variables $\mathbf{x}_{1}$ and $\mathbf{x}_{2}$ with SNP coding $\left\{ 0,1,2 \right\}$. A greedy, sequential algorithm is often used to find the best sequence of the binary partitions. It starts with all data, and then selects splitting variable $x_{j}$ and split point $\tau_{\eta}$ that solve

$\min\left[ \min\sum_{i\in r_{1}\left( x_{j},\tau_{\eta} \right)} \left( y_{i}-\hat{\mu}_{1} \right)^{2}+\min\sum_{i\in r_{2}\left( x_{j},\tau_{\eta} \right)} \left( y_{i}-\hat{\mu}_{2} \right)^{2} \right]$, (1)

where $\hat{\mu}_{1}=\mathrm{ave}\left( y_{i}\left| x_{ij} \right.\in r_{1}\left( x_{j},\tau_{\eta} \right) \right)$ is the mean for region $r_{1}$ and $\hat{\mu}_{2}=\mathrm{ave}\left( y_{i}\left| x_{ij} \right.\in r_{2}\left( x_{j},\tau_{\eta} \right) \right)$ is the mean for region $r_{2}$. This is also known as a piecewise constant regression function. In the example, $x_{1}$ is the first chosen variable and the first splitting point is at $x_{1}=0$, which means that $x_{1}=\left\{ 1,2 \right\}$ forms the other partition. This partition is illustrated as the top split in the regression tree in Figure 1a. Having determined the best split, the data is partitioned into the two resulting regions and the algorithm continuous with further splitting in each of the regions based on the second most informative variable, or stops if some stopping criteria is met. It turns out that $x_{2}=0$ provides the best splitting point in $r_{1}\left( j_{1},\tau_{1} \right)$ and eventually this results in the first terminal node $\mu_{1}=-1.0$. The remaining region of this split is further partitioned into two regions based on $x_{2}=1$ and $x_{2}=2$ that result in leaf nodes $\mu_{2}=-0.7$ and $\mu_{3}=-0.9$. The right main branch of the tree is first partitioned into $x_{1}=1$ and $x_{1}=2$, and it turns out that $x_{1}=1$ results in terminal node $\mu_{4}=0$ since $x_{2}$ doesn’t contribute any further splits here. On the other hand, when $x_{2}$ is conditioned on $x_{1}=2$, this region is split into the three terminal nodes $\mu_{5}=1.0$, $\mu_{6}=0.7$ and $\mu_{7}=0.9$ resulting in the final tree in Figure 1a and the leaf node surface in Figure 1b.

The interesting property of this approach is that it has the capacity to describe all the traditional genetic effects in a joint framework. By looking at the terminal node surface (Fig. 1b), we see that variable $x_{1}$ mostly describes an additive genetic effect, i.e. a more or less linear effect on the response over all values of $x_{2}$. Variable $x_{2}$ shows different patterns depending on the value of $x_{1}$, which means that there is a complex interaction effect between these two variables (in genetic terminology corresponding to epistasis). When $x_{2}$ is conditioned on $x_{1}=0$, the highest leaf node value is obtained for the heterozygote $x_{2}=1$ which indicates over-dominance. In contrast, when $x_{2}$ is conditioned on $x_{1}=2$, the lowest leaf node value is achieved for the heterozygote $x_{2}=1$ which is the same as under-dominance. Hence, regression trees can easily describe complicated higher order interactions that is not feasible with the traditional linear model genetic effects approach.

In situations with more than two predictor variables, the regression tree possibly will include several variables. The number of potential splits quickly increases with the number of predictors and it will be important to control the size of the tree. Several approaches for growing trees have been proposed. Hastie *et al*. [2] suggest a strategy where a large tree $\mathbb{T}_{0}$ is grown until a minimum node depth of at least 5 is reached. This tree is then pruned based on cost-complexity pruning. Pruning is the process where the internal nodes are collapsed which results in a set of smaller subtrees $\mathbb{T}_{S}$. The cost-complexity criterion is defined as

$C_{\lambda}\left( \mathbb{T}_{S} \right)=\sum_{r=1}^{\left| \mathbb{T}_{S} \right|} N_{r}Q_{r}\left( \mathbb{T}_{S} \right)+\lambda\left| \mathbb{T}_{S} \right|$ (2)

where $\left| \mathbb{T}_{S} \right|$ is the number of terminal nodes in $\mathbb{T}_{S}$, $N_{r}$ the number of predictor variables in region *r*, $Q_{r}\left( \mathbb{T}_{S} \right)$ the sum of squares of region *r* divided by $N_{r}$, and $\lambda$ is a regularization parameter that needs to be tuned because it determines the tradeoff between tree size and goodness of fit to the data. Large values of $\lambda$ give small trees and small values of $\lambda$ large trees. The goal is to find the tree that minimize $C_{\lambda}\left( \mathbb{T}_{S} \right)$ by successively collapsing internal nodes from the bottom and up over different values of $\lambda$ (i.e. weakest link pruning). The optimal $\lambda$ can be obtained with cross-validation.

Figure 1a. Example of a regression tree resulting from the successive binary splits of two SNP variables ($x_{1}$ and$x_{2}$) applied to a toy phenotype response **y**. The regression tree is fully described by the tree structure, the split rules and the seven terminal nodes $\mu_{i}$.


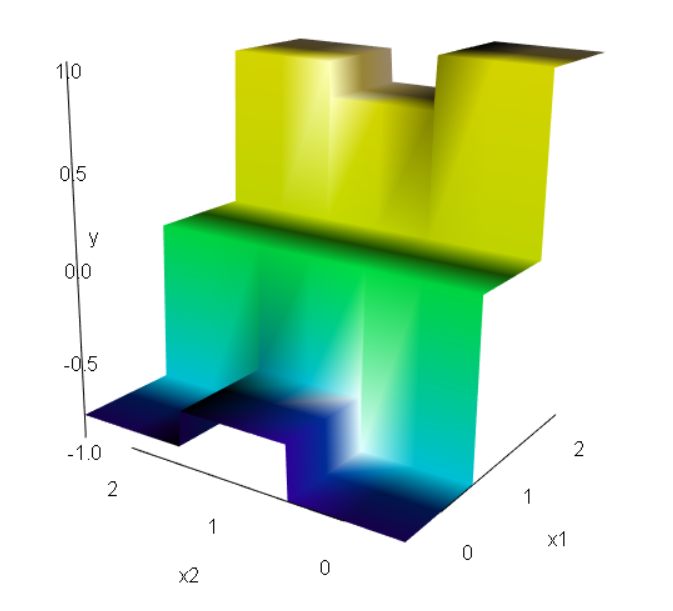


Figure 1b. The response surface resulting from the regression tree in Fig. 1a. Note that SNP $x_{1}$ shows an overall linear additive genetic effect, whereas the effect of SNP $x_{2}$ describes over-dominance, no effect or under-dominance conditional on the genotype of SNP $x_{1}$.
